# The Senescence-Associated Secretory Phenotype (SASP) in the Challenging Future of Cancer Therapy and Age-Related Diseases

**DOI:** 10.3390/biology9120485

**Published:** 2020-12-21

**Authors:** Lorenzo Cuollo, Fabrizio Antonangeli, Angela Santoni, Alessandra Soriani

**Affiliations:** 1 Department of Molecular Medicine, Sapienza University of Rome, Laboratory Affiliated to Istituto Pasteur Italia-Fondazione Cenci Bolognetti, 00161 Rome, Italy; lorenzo.cuollo@uniroma1.it (L.C.); angela.santoni@uniroma1.it (A.S.); 2 Center for Life Nano Science, Sapienza, Istituto Italiano di Tecnologia, 00161 Rome, Italy; 3 Institute of Molecular Biology and Pathology, National Research Council (CNR), 00185 Rome, Italy; fabrizio.antonangeli@uniroma1.it; 4 IRCCS (Istituto di Ricovero e Cura a Carattere Scientifico) Neuromed, 86077 Pozzilli, Italy

**Keywords:** senescence, SASP, inflammation, cancer therapy, age-related disease, senolytic, senomorphic

## Abstract

**Simple Summary:**

A fundamental feature of cellular senescence is the emergence of the Senescence-Associated Secretory Phenotype (SASP), which represents a considerable source of inflammatory and tissue-remodeling cues. The pathophysiological relevance of senescence and SASP has generated a fertile area of research aimed at manipulating the SASP to fight cancer and age-related conditions. This review enlightens the most important mechanisms that regulate the SASP and summarizes the current evidence on the feasibility of intervening on its composition, providing a reading frame of the general potentialities of SASP modulation.

**Abstract:**

Cellular senescence represents a robust tumor-protecting mechanism that halts the proliferation of stressed or premalignant cells. However, this state of stable proliferative arrest is accompanied by the Senescence-Associated Secretory Phenotype (SASP), which entails the copious secretion of proinflammatory signals in the tissue microenvironment and contributes to age-related conditions, including, paradoxically, cancer. Novel therapeutic strategies aim at eliminating senescent cells with the use of senolytics or abolishing the SASP without killing the senescent cell with the use of the so-called “senomorphics”. In addition, recent works demonstrate the possibility of modifying the composition of the secretome by genetic or pharmacological intervention. The purpose is not to renounce the potent immunostimulatory nature of SASP, but rather learning to modulate it for combating cancer and other age-related diseases. This review describes the main molecular mechanisms regulating the SASP and reports the evidence of the feasibility of abrogating or modulating the SASP, discussing the possible implications of both strategies.

## 1. Introduction

The Senescence-Associated Secretory Phenotype (SASP), or Senescence-Messaging Secretome (SMS), can be defined as a highly variable, dynamic, and long-lasting program of senescent cells, consisting in the abundant secretion of generally proinflammatory compounds in the tissue microenvironment [1]. Investigators of SASP have demonstrated the presence and the biological relevance, among the secreted proteins, of numerous cytokines, growth factors, chemokines, and matrix-metalloproteinases. Moreover, the contribution of small molecules, such as ROS, miRNAs, and extracellular vesicles (EVs), which represent an intensively investigated area of research, may be considered an important target in the future [2,3].

The spectrum of secreted molecules seems to be so broad, diversified, and context-dependent that, unsurprisingly, contradictory interpretations have been proposed about the role of SASP in the pathophysiology of chronic diseases [4]. Albeit the general agreement on the detrimental aspects of the SASP in the context of cancer and age-related disorders, the evidence for a protective role of SASP-evoked immune response should not be neglected [5]. Indeed, the SASP has the potential to attract innate and adaptive immune cells in proximity of tumor cells and pre-malignant lesions [6,7,8] or enhance cytotoxicity against drug-induced senescent tumor cells [9,10,11,12].

The experimental models used to investigate senescence and SASP still confirm the intricacy of the subject. Certainly, the type and strength of the senescence-inducing stimulus, the identity of the cell undergoing senescence, the composition, and time-dependent variability of the secretome, are all crucial aspects to consider in the research on SASP. In addition, when evaluating the effects of SASP within tissues or tumors, the quality of the immune infiltrate and the persistence/accumulation of senescent cells over time represent additional layers of complexity. However, a certain degree of overlap has been demonstrated among various SASPs, with specific proteins being found almost invariably, namely IL-1, IL-6, IL-8, GROα/β, GM-CSF, MMP-1, MMP-3, MMP-10, ICAM-1, PAI-1, and IGFBPs [13].

## 2. Regulation and Modulation of SASP: An Overview

### 2.1. Perplexities and Potentialities

Currently, parallel lines of research are aiming at selectively eliminating senescent cells with the use of senolytics, or preserving the cytostatic effect of senescence while abrogating the secretory phenotype (senomorphics). Both these approaches lie on solid evidence of the negative impact of senescence and SASP in models of chronic diseases. Indeed, the seminal work of Baker and colleagues (2011) demonstrated how the selective elimination of senescent cells (or at least of p16^Ink4a^-expressing cells) in BubR1 hypomorphic mice results in delayed onset of age-related conditions [14]. Abrogation of SASP in senescent preadipocytes using JAK inhibitors similarly reduces systemic inflammation and frailty in aged mice [15]. Moreover, ablation of the proinflammatory secretome of premalignant senescent cells by deletion of IL-1α gene leads to a lesser number of neoplastic lesions in a mouse model of pancreatic cancer [16]. 

On the other hand, senescent cells harbor the potential to locally reawaken the immune system, sometimes with desirable outcomes. To this regard, it has been shown that premalignant senescent hepatocytes secrete chemokines and cytokines that mediate their clearance by CD4^+^ T cells [17]. 

Intercellular communication is essential for both the physiological and pathological effects of senescent cells on the microenvironment. As an example, senescent fibroblasts transfer proteins to Natural Killer (NK) cells by a mechanism depending on cell-cell contact and CDC42-regulated actin polymerization, promoting NK cell activation and cytotoxicity [18].

It appears that the SASP can either inhibit or promote tumor development depending on the status of p53. In the liver, senescent stellate cells secrete factors that skew macrophage polarization towards tumor-inhibiting M1 state. In contrast, p53-null stellate cells secrete factors that promote a M2 pro-tumorigenic phenotype and increase the proliferation of premalignant cells [19]. In a model of transferred liver tumor cells the induced expression of p53 triggers senescence and leads to the production of various chemokines, including CCL2, which is essential for robust infiltration of NK cells in the tumor [20].

**Figure 1:**
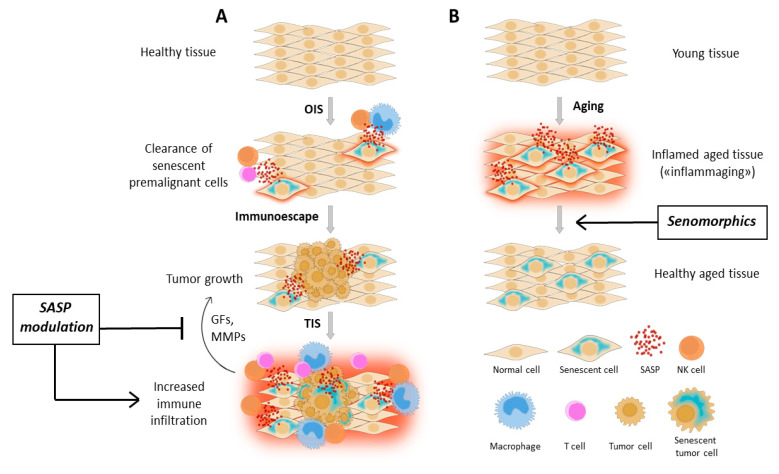
Therapeutic approaches of Senescence-Associated Secretory Phenotype (SASP) modulation (**A**) SASP modulation could synergize with therapy-induced senescence (TIS), abrogating the pro-tumorigenic properties of SASP while preserving or even boosting the anti-tumor immune response; (**B**) the use of senomorphics in age-related disorders could abolish the chronic and sterile inflammation that causes tissue disfunction. Abbreviations: SASP: Senescence-associated secretory phenotype; OIS: Oncogene-induced senescence; TIS: Therapy-induced senescence; GFs: Growth factors; MMPs: Matrix metalloproteinases.

Despite the controversy, the research on cellular senescence is slowly moving towards the modulation of secretome composition, with the hope of controlling or even take advantage of this powerful source of bioactive molecules. The aims are fairly ambitious, and in some way conflicting: (I) To turn senescent cells into allies of the immune system against cancer. To achieve this goal, SASP should be finely tailored in order to suppress the production of any growth factor or matrix-remodeling enzyme that could be advantageous for growth and spreading of tumor cells, preserving, or enhancing the activity of chemokines and cytokines that attract and stimulate the immune system in loco. This scenario could be considered as a new type of immunotherapy, potentially able to synergize with the classical therapy-induced senescence (TIS). (II) to abrogate the sterile inflammation caused by the accumulation of senescent cells in the tissues, which undoubtedly contributes to numerous age-related pathologies, without renouncing the intrinsic tumor-protecting and pro-regenerative nature of senescence. The compounds able to achieve this effect have been called “senomorphics”, in opposition to senolytics (Figure 1). 

Indeed, extensive research has finally revealed that the proliferative arrest and the establishment of SASP are distinct phenomena regulated by different pathways. For instance, L. Lau and colleagues demonstrated the possibility of uncoupling cell cycle exit and SASP by intervening on IL-1/IL-1R signaling. Further, the appearance of other typical markers of senescence, such as the Senescence-Associated β-Galactosidase (SAβ-Gal) activity, decreased BrdU incorporation, Senescence-Associated Heterochromatin Foci (SAHF), and Cytoplasmic Chromatin Fragments (CCFs), seem to be dissociated from the emergence of the proinflammatory secretome. Remarkably, these observations are valid for both RAS- and etoposide-induced senescence [16].

In order to intervene on the senescent secretome, the precise understanding of the molecular pathways underlying its variability in composition and time-dependency will be of utmost importance in the future of research. The following section will discuss the most important actors that control the establishment of SASP; however, the main focus of the present review is the artificial modulation of the secretome and its implications. For more comprehensive dissertations on the pathways underlying SASP, we refer to excellent reviews on the subject [21,22,23].

### 2.2. Molecular Mechanisms Regulating SASP

The mechanisms behind the establishment and variability of SASP seem remarkably complex and are still incompletely understood; however, it is certainly known that SASP is regulated at multiple levels, including transcription, translation, mRNA stability, and secretion. It is also clear that SASP relies on autocrine and paracrine positive-feedback loops that cause robust signal amplification [24]. 

Persistent DNA-Damage Response (DDR) signaling, when it does not culminate in regulated cell death, “locks” cells in a senescent state and leads to development of senescence-associated phenotypes, including the SASP [25,26]. However, the DDR cannot be the sole regulator, since transient DNA damage does not induce senescence and SASP. In addition, the DDR is activated shortly after the damage, whereas the SASP usually develops several days later [26,27]. 

NF-κB and CCAAT/enhancer-binding protein (C/EBPβ) complexes, both being transcriptional machineries associated with inflammatory responses, are currently recognized as the main transcription factors globally regulating SASP expression [28,29].

There is a strong correlation between DNA damage-induced senescence and NF-κB-regulated SASP [26,30]. In response to genotoxic stress, Ataxia telangiectasia mutated (ATM) kinase activates NF-κB through post-translational modifications (PTMs) of NEMO that are critical for NF-κB activation [31,32]. ATM activates NEMO, which in turn activates the IKK complex, resulting in nuclear translocation of NF-kB and transcription of several SASP-related genes [33]. In melanoma, senescent cells produce a secretome that displays pro-invasive and pro-tumorigenic properties and that depends on PARP-1 and NF-κB [34].

The expression of SASP components IL-6 and IL-8 requires IκBζ in both DNA damage- and oncogene-induced senescence (OIS), revealing IκBζ as an essential regulator of proinflammatory SASP [35].

Many lines of evidence demonstrate that sustained DDR is required for robust SASP production since the depletion of DDR components, such as ATM, NBS1, or CHK2, inhibits the expression of IL-6, IL-8, and several GRO family members. Accordingly, it has been demonstrated, at least in some experimental settings, that the activation of DDR, but not the presence of DNA damage per se, regulates the senescent states and SASP [36,37]. 

New findings strongly suggest that the cytosolic DNA sensor cGAS links DDR to SASP initiation. In response to the accumulation of cytoplasmic DNA (CCFs, mtDNA) cGAS synthesizes the second messenger 2’3’-cGAMP, which activates the adaptor protein STING, an activator of both IRF3 and NF-κB [38,39]. Free cytosolic DNA is considered the main initiator of this pathway, and micronuclei are believed to be its major source. The nucleolar acetyltransferase NAT10 is involved in micronuclei formation and induces SASP in colorectal cancer cells, whereas its chemical inhibition or genetic deletion markedly reduces micronuclei formation and, consequently, SASP emergence [40]. 

Previous work demonstrated that loss of lamin B1, a structural protein of the nucleus, occurs when cells undergo senescence by diverse stimuli. This decrease, now recognized as a novel marker of senescence, is independent of DDR, p38 MAPK, NF-κB, and reactive oxygen species (ROS) but depends on direct stimulation of the p53 or pRB pathway [41]. Loss of lamin B1 seems to be strictly connected with the activation of cGAS and the subsequent production of inflammatory SASP components, since cGAS can recognize aberrant CCFs arising as a consequence of nuclear lamin B1 degradation in senescent cells [42]. 

Surprisingly, CCFs can also be formed due to dysfunctional mitochondria triggering ROS-JNK signaling. The SASP is then established through the subsequent activation of the cGAS-STING pathway [43].

NF-κB activation upon DNA damage could be also mediated by a non-canonical pathway that involves ATM and PARP-1, as well as the DNA sensing factors IFI16 and STING, but is independent of cGAS and 2’3’-cGAMP production [44]. 

Senescence-associated NF-κB activation has been also linked to mitogen-associated protein kinases (MAPKs) [23,45,46]. Among the three main classes of MAPKs, ERK1/2 (extracellular signal-regulated kinase) and p38 have been most closely linked to cellular senescence [25,47,48,49,50,51]. Indeed, p38 MAPK is important to sustain cell cycle arrest by activating p53 and pRB/p16/CDKN2A pathways [49,52]. p38 activity is necessary and sufficient for SASP development in DNA-damage or RAS-induced senescent cells, mainly through NF-κB transcriptional regulation, which in turn promotes the expression of proinflammatory genes [26,49,53].

While its capacity to induce senescence is undisputed, the role of p53 in SASP regulation is more controversial. Indeed, p53 may be a negative regulator of the SASP, since its inactivation amplifies the SASP in part by enhancing p38MAPK-mediated activation of NF-κB [49]. In another report, the loss of function of p53 in multiple cellular models resulted in amplification and faster development of SASP after X-irradiation [54]. On the contrary, the group of S. Lowe, using a mosaic mouse model of liver carcinoma, clearly demonstrated that reactivation of p53 in p53-deficient tumor cells results in senescence onset and production of a proinflammatory SASP, leading to the recruitment of NK cells and tumor clearance [8]. Additional studies are needed to explain these contradictory results; the explanation might not be solely related to the diversity of models, but also to the numerous post-translational modifications of p53 [55].

A crosstalk mechanism between MAPKs and RNA-binding proteins (RBPs) in the regulation of a plethora of biological phenomena has been unraveled [56]. In particular, RBPs such as AUF1, HuR, hnRNPA1, GRSF1, and TTP have been linked to the expression of many SASP factors [57,58,59,60,61]. hnRNPA1 can suppress SASP induction by SIRT16-mediated deacetylation of NF-κB [61]. MK2 causes dissociation and stabilization of IL-6, IL-8, and IL-1B mRNAs through ZFP36L1 and AUF1 phosphorylation in senescent cells [57,62]. The RBPs HuR regulates not only the replicative lifespan, but also the expression of NF-κB-regulated SASP factors in mouse fibroblasts [58]. 

Interestingly, the scavenger receptor CD36 seems to be strictly required for NF-κB phosphorylation and SASP initiation/maintenance during both oncogene- and chemical-induced senescence [63]. 

The transcription factor GATA4 accumulates during IR-induced, OIS, and replicative senescence and activates a relevant portion of senescence-associated genes. This pathway is also dependent on the DDR kinases ATM and ATR, which can block p62-dependent autophagic degradation of GATA4 [64]. In human mesenchymal stem cells, GATA4 regulates SASP activation caused by progerin, a truncated mutant form of lamin A protein [65].

Many of the mechanisms discussed so far regulate SASP by converging on NF-κB; however, the transcription factor C/EBPβ has been proved to be critical for SASP as well. C/EBP family comprises transcription factors that are part of the basic leucine zipper (bZIP) super-family [66]. Cells undergoing OIS up-regulate C/EBPβ and secrete several CXCR2-binding chemokines in a manner dependent on both NF-κB and C/EBPβ; the latter then cooperates with IL-6 to amplify the activation of the inflammatory network, which includes IL-8 [28,29]. Curiously, C/EBPβ is inhibited by the 3′ untranslated region (3′UTR) of its own mRNA, which impairs the cytostatic and pro-senescence functions of C/EBPβ selectively in immortalized and transformed cells [67]; the C/EBPγ isoform participates in the regulation of C/EBPβ neutralizing its cytostatic activity and suppressing the basal transcription of SASP genes through heterodimerization [68]. 

More recently, a zinc finger transcription factor, named Zscan4 (Zinc Finger and Scan Domain Containing 4), was found to be essential for SASP development in human stromal cells exposed to acute stress upon anti-cancer treatments [69].

Alterations in chromatin modifications, such as H3K9me2, have been implicated in regulating SASP gene expression [70]. For instance, it was demonstrated that the histone variant macroH2A1 is required for the senescence-associated persistent DDR and is a critical control point in the transcriptional regulation of SASP genes [71]. 

Finally, it is now largely accepted the role of ncRNAs (microRNAs, lncRNAs, circRNAs) in the regulation of SASP genes transcription, mRNAs stability, and translation and/or secretion of SASP proteins, although the knowledge on circRNAs is still at early stages [72].

The control of SASP by microRNAs often involves the regulation of NF-kB. In a model of senescent fibroblasts, Bhaumik and colleagues demonstrated a negative role for miRNA146a/b in the secretion of IL-6 and IL-8 through the control of IL-1 receptor-associated kinase 1 (IRAK1), which in turn is responsible of NF-κB activation [73,74].

A wide variety of miRNA can be encapsulated within exosomes, which have been recently included as part of the SASP. Extracellular microRNAs present in body fluids may interact with Toll-like receptors (TLRs) thus interfering with the production of many SASP factors. In human macrophages, miR-21 functionally interact with TLR8 in endosomes leading to the secretion of cytokines such as TNF-α and IL-6, through NF-κB activation [75]. miR-155, controlling the expression of IKKβ and IKKε, may lead to NF-κB repression controlling SASP and aging-associated inflammation [74]. Similarly, miR-199a affects NF-κB activity in ovarian cancer cells by controlling IKKβ expression [76]. miR-34a overexpression by replicative-senescence on primary human aortic smooth muscle cells (HASMCs) stimulates the induction of pro-inflammatory factors [77].

The expression of the TLR ligands-dependent lncRNA LincRNA-Cox2 is linked to the repression of the transcription of several proinflammatory genes by interacting with heterogeneous nuclear ribonucleoprotein (HNRNP) A/B and A2/B1 [78]. The human long noncoding RNA, lnc-IL7R, regulates LPS-induced proinflammatory mediators, such as IL-8, IL-6, E-selectin, and VCAM-1 [79]. Lethe lncRNA may act as a repressor of NF-κB activity by binding directly to the RelA homodimer, blocking the ability of RelA to bind DNA and leading to a decreased production of pro-inflammatory cytokines [80].

### 2.3. Impact of Senescent Cell’s Metabolism on SASP

The composition of SASP is heavily influenced by the peculiar metabolism of the senescent cell, albeit investigators just started to scratch the surface of this deep and intricate field [81].

Loss of mitochondrial homeostasis is crucial for induction of senescence and can determine the quality of SASP. Depletion of mitochondrial SIRT3 and SIRT5 induces senescence and, more interestingly, abrogates the secretion of IL-1β, CXCL1, CXCL2, IL-6, IL-8, and VEGF by IR-induced senescent human fibroblasts. Mitochondrial dysfunction caused by other means similarly induces a senescent arrest characterized by a peculiar SASP, overlapping but different from the SASP induced by conventional senescence inducers like genotoxic stress or oncogene activation. A decreased NAD+/NADH ratio seems to be responsible for this Mitochondrial Dysfunction-Associated Senescence (MiDAS) through the AMPK-p53 axis, which negatively affects NF-κB activity [81]. SIRT3 and SIRT5 are therefore novel potential targets for anti-inflammatory SASP modulation. 

The fall of NAD+ levels could also dampen the activity of PARP, a key enzyme involved in the nuclear DNA repair machinery and mitochondrial DNA integrity, which uses NAD+ as an ADP-ribose donor [82,83]. A PARP-1/NF-κB signaling cascade drives the establishment of a pro-tumoral and pro-metastatic SASP in a melanoma xenograft model [34], though the precise role of NAD+ levels in this context is unknown.

## 3. SASP Modulation by Genetic and Pharmacological Targeting

Considering the deep influence of SASP on tissue homeostasis, especially in the context of anti-tumor therapy and aging, the possibility of altering the composition and timing of the senescence-associated secretome, is of particular interest. Different approaches have been carried out so far; they all provide a proof of concept of the feasibility of reprogramming or abrogating SASP in vivo to promote anti-tumor immune response or contrast the negative impact of “inflammaging”.

In a mouse model of conditional *Pten*-null prostate cancer, Toso and colleagues discovered that STAT3 inactivation, either by genetic knockout or pharmacological JAK2 (an upstream activator of STAT3) inhibition, modifies SASP composition without affecting the establishment of senescence and the subsequent cell growth arrest [7]. In particular, the targeting of STAT3 reduces the secretion of chemokines involved in myeloid-derived suppressor cell (MDSC) polarization, such as CXCL2, GM-CSF, M-CSF, IL-10, and IL-13, without altering the levels of CCL2 (MCP-1) and CXCL10 (IP-10), leading to improved recruitment of CD8^+^ T cells and NK cells at the tumor site. Remarkably, as discussed by the authors, the SASP composition strictly depends on the hit triggering cellular senescence and on the array of transcription factors activated, with OIS characterized by lower expression of STAT3 compared to *Pten*-loss-induced cellular senescence. This aspect should be carefully considered before approaching therapies aimed at modifying SASP by STAT targeting.

The possibility of SASP modulation by targeting the JAK/STAT pathway has been further addressed by treating human primary senescent preadipocytes (also called fat cell progenitors) with different JAK inhibitors [15]. JAK inhibition reduces the secretion of GM-CSF, G-CSF, CXCL1, IL-6, IL-8, and several others inflammatory SASP proteins specifically in senescent cells; this effect has been linked to decreased systemic and adipose tissue inflammation, accompanied by enhanced physical activity in aged mice. Surprisingly, SASP components such as IL-1α/β, IL-7, IL-15, IFN-γ, Fractalkine, FGF-2, EGF are not significantly affected by JAK inhibition.

As stated above, a large body of evidence points out cGAS-STING pathway as central in the establishment of senescence and SASP [39,42]. Indeed, upon sensing of cytoplasmic DNA, cGAS promotes both IRF3 and NF-κB activity through TANK-binding kinase-1 (TBK1) leading to senescence and SASP establishment [38,84,85]. Accordingly, genetic depletion or chemical inhibition (by treatment with epigallocatechin gallate) of the factor Ras GTPase activating protein-binding protein 1 (G3BP1), which acts upstream of cGAS, impairs the association of the latter with cytoplasmic DNA, resulting in reduced phosphorylation of IκBα and STAT3 [86]. These events abolish the SASP, as evaluated by IL-6 and IL-8 levels, without compromising cell commitment to senescence. Interestingly, G3BP1 knockdown is associated with Lamin B1 loss, a newly discovered marker of cellular senescence [41,86]. 

The transcription factor NF-κB represents a promising target for SASP control. It has been reported that cortisol in humans and corticosterone in rodents reduce the DNA binding activity of NF-κB by dampening the IL-1α signaling [87]. Consequently, several NF-κB-dependent proinflammatory SASP factors are downregulated, including IL-6, IL-8, GM-CSF, and IL-1α itself. IL-1α, activating NF-κB, is a key upstream regulator of the SASP and, at the same time, is an NF-κB transcriptional target [88]; this positive feedback loop is quenched by glucocorticoids.

Metformin is an anti-diabetic drug with pleiotropic effects that is also active on senescent cells [89]. Among its effects, metformin impairs the SASP of RAS-induced senescent cells without impeding the proliferative arrest, by inhibiting IKKα/β and IκB phosphorylation and preventing p65 (RelA) nuclear translocation [90]. Metformin acts negatively on NF-κB without affecting other inflammatory pathways such as p38, JNK, and IRF. Metformin-mediated inhibition of SASP may contribute to the anti-aging effects observed after metformin treatment [91,92].

The idea of targeting NF-κB as a general strategy to counteract inflammatory pathologies and NF-κB-related cancer resistance to therapy is emerging [93,94,95]. IKK inhibitors are already available and could be tested for SASP modulation. Nevertheless, several concerns exist regarding the systemic administration of NF-κB inhibitors due to their off-target pleiotropic effects [94,95]. It is reasonable that feasible approaches will include intermittent administration and usage in combined therapies.

The pivotal role played by IL-1α in SASP maintenance is highlighted by the use of rapamycin, a selective inhibitor of the mTOR complex 1 (mTORC1). Indeed, rapamycin can modulate the SASP by reducing the translational efficiency of *IL-1α* mRNA [96]. After rapamycin treatment, low levels of IL-1α are bound to the cell surface, leading to a blunted IL-1R1 signaling. Consequently, NF-κB-dependent expression of IL-6 and IL-8 is suppressed in senescent cells. Targeting IL-1/IL-1R axis may represent a valid strategy to downmodulate the late proinflammatory arm of SASP controlled by NF-κB without altering the proliferative arrest, which is generally considered beneficial [16].

mTOR itself impacts on SASP at several levels. The eukaryotic translation initiation factor 4E-binding protein 1 (4EBP1), a well-known target of mTOR, is able to control the translation of specific mRNAs involved in SASP regulation [62]. The mitogen-activated protein kinase-activated protein kinase 2 (MAP-KAPK2 or MK2) is located downstream of p38 and is activated in senescent cells [49]. Inhibition of mTOR (by different inhibitors such as rapamycin, Torin 1, and NVP-BEZ235) decreases the 4EBP1-dependent MAP-KAPK2 mRNA translation leading to a reduced phosphorylation of its targets. Among those, the Zn-finger protein ZFP36-L1, in the unphosphorylated status, targets the mRNAs of several SASP-related cytokines for degradation. Hence, mTOR inhibition strongly impairs SASP and reduces inflammation. 

Moreover, ATM, AKT, and mTORC1 phosphorylation cascade integrates signals from DDR and promotes PGC-1β-dependent increase in mitochondrial mass, which in turn contributes to cellular senescence and secretion of proinflammatory IL-6, IL-8, GRO, and MCP-1 [97].

Complete abrogation of SASP is achieved by the killing of senescent cells through senolytic drugs or immune-mediated strategies, however a dissertation on these alternative approaches is beyond the scope of the present review and we refer to comprehensive reviews on the topics [98,99,100]. Briefly, dasatinib, a pan-tyrosine kinase inhibitor used as anti-cancer drug, and quercitin, a natural flavonoid, have been employed as senolytic agents in research models and human clinical trials reporting reduced senescence signature [101,102,103]. BCL inhibitors (such as ABT-263 and ABT-737), as well as HSP90 inhibitors (geldanamycin and 17-DAMG), have been used to overcome the anti-apoptotic and pro-survival features of senescent cells, respectively [104,105,106]. It should be taken into account that, as senescent cells participate in placental development, embryogenesis, and wound healing [50,107,108,109], the widespread removal of senescent cells could lead to unexpected effects.

Since chronic inflammation can contribute to pro-tumorigenic effects and organ damage, but it is also required for the senescence-associated immune surveillance, the timing of SASP-targeting therapies, in respect to tumor stage or immunological context, may have a deep influence on the outcome of the aforementioned strategies.

As a matter of fact, the time-dependent variation of SASP appears to be critical for the interaction with the immune system and, consequently, for the fate of senescent cells in the tissue. For instance, the group of M. Narita has recently demonstrated how Notch signaling constitutes a switch between an early TGF-β-enriched secretome and a late C/EBPβ-mediated proinflammatory secretome, typically characterized by IL-1, IL-6, and IL-8. Notch1 transient activation proved to suppress the function of C/EBPβ, negatively regulating the production of inflammatory cytokines in the early phase of senescence. Consistently, inhibition of Notch accelerates the clearance of senescent hepatocytes in a mouse model of RAS-induced senescence, mainly through an increased CD4+ T-cell recruitment [6].

Using a large-scale siRNA approach, Georgilis and colleagues identified targetable SASP modulators that do not revert the senescence growth arrest, focusing on the polypyrimidine tract binding protein 1 (PTBP1) [110]. PTBP1 is an alternative splicing factor regulating the exon skipping of EXOC7, a protein involved in intracellular trafficking and exocytosis [111]. PTBP1 depletion downmodulates NF-kB pathway and impairs IL-1α, IL-6, and IL-8 secretion in various cell types exposed to different senescence-inducing stimuli. In addition, it abrogates the inflammatory effects of SASP, reducing immune cell infiltration in mouse models of liver cancer. Notably, in the investigated tumor models, the decreased immunosurveillance was not associated with an increased tumorigenesis [110]. 

Senescent cells undergo extensive chromatin remodeling, and the occupancy of bromodomain protein BRD4 to senescence-activated super enhancers flanking SASP-related genes has been observed during OIS [112]. Accordingly, BET inhibitor JQ1 blunts SASP affecting, among others, IL-1α, IL-1β, IL-6, and IL-8 production. Consequently, the paracrine senescence induction is dampened. During senescence, BRD4 signature partially overlaps with that of NF-κB, as expected by two key regulators of the SASP, but also covers unique genes, i.e., *INHBA*, *BMP2*, *VEGFA*, and *VEGFC*. Remarkably, BRD4 inhibition leads to a reduced NK cell-mediated immunosurveillance of senescent cells [112]. Other chromatin modifiers, namely mixed-lineage leukemia protein 1 (MLL1) and high-mobility group box 2 (HMGB2), reshape the epigenetic landscape of senescent cells towards an inflammatory SASP-dependent phenotype and in accordance with this function their inhibition repress inflammation without blocking senescence [113,114].

Studies carried out so far have placed particular emphasis on IL-6 and IL-8 secretion for evaluating the effects of SASP modulation. More generally, the study of secretome has been often focused on proinflammatory cytokines and chemokines, while the biological effects of other components of SASP have been less investigated, including tissue remodeling factors and growth factors, which are presumably important for tissue regeneration. This may represent a caveat in evaluating the long-term effects of SASP ablation in vivo. Furthermore, it should be noted that ablation of proinflammatory cytokines does not necessarily implicate the ablation of other signals. It would be desirable to adopt a more unbiased approach when evaluating the effect of senomorphics and genetic manipulation on SASP composition, by extending the analysis to anti-inflammatory molecules, signaling proteins acting on non-immune cells, small molecules, and EVs.

On the other hand, despite the increasing efforts to characterize in detail the secretome of specific experimental models using high-throughput methods, warranties are needed before accepting general conclusions, as SASP composition mirrors, at least in part, cell type-specific pathophysiology, creating each time a peculiar setting that should be considered. SASP impairment with reduced levels of inflammatory cytokines could in principle lead to opposite effects, decreasing the immunosurveillance performed by type 1 macrophages and cytotoxic lymphocytes (T and NK cells) or preventing the recruitment of immune-suppressive pro-tumorigenic cells such as MDSCs, depending on the local immune landscape and tissue microenvironment. Moreover, upon SASP modulation it is not rare to observe a strong reduction in the levels of a subset of chemokines, while others remain largely unaffected; it would be interesting to study the subsequent time-dependent variation of the immune landscape and its repercussions.

The research on SASP and its modulation in cancer has largely employed RAS-induced senescence or senescence caused by loss of function of tumor suppressors (p53, PTEN) as models of choice (Table 1). This should add a note of caution in generalizing the results obtained so far. Indeed, the most common form of senescence during clinically relevant stages of cancer is, predictably, Therapy-Induced Senescence (TIS) provoked by either anti-tumor drugs or ionizing radiation, whose effects are much more widespread and destructive. It is not necessarily expected that comparable results could be obtained in models of TIS, in which multiple forms of cellular stress (genotoxic, oxidative, metabolic, etc.) arise simultaneously and, presumably, relay on a greater variety of signaling pathways. 

## 4. Conclusions

Cellular senescence and SASP have great potential as immunomodulatory tools for their intimate connection with the immune system. However, joint efforts will be needed for deciphering and manipulating such intricate network. 

To date, excellent works provided evidence of the possibility of exploiting SASP against cancer or abolishing it with senomorphics to prevent the consequences of chronic inflammation. Both approaches seem remarkably promising and for sure will positively impact the future of research on senescence. Nevertheless, the adoption of more various and realistic models of cellular senescence is urgently needed. Finally, a more unbiased approach in the analysis of the secretory activity will hopefully provide additional clues to interpret the function and the significance of senescent cells in disease.

## Figures and Tables

**Table 1:** Experimental models used to study SASP modulation.

Cell Type	Experimental Model of Senescence	Target of SASP Modulation	References
human epithelial cell	Pten^loxP/loxP^	STAT3	[7]
human pre-adipocytes	ionizing radiation	JAKs	[15]
human fibroblasts	ionizing radiation/H-ras V12	G3BP1	[86]
human fibroblasts	H-ras V12	IKKα/β;IκB	[90]
human fibroblasts	ionizing radiation/H-ras V12	mTORC1	[96]
human fibroblasts	H-ras V12	IL1/IL-1R	[16]
human fibroblasts	H-ras V12	4EBP1	[62]
human fibroblasts	H-ras V12	NOTCH1	[6]
MR90, MCF7 and HFFF2	H-ras V12	PTBP1	[110]
human fibroblasts	H-ras V12	BRD4	[112]
